# Caspase-1/IL-1β represses membrane transport of GluA1 by inhibiting the interaction between Stargazin and GluA1 in Alzheimer’s disease

**DOI:** 10.1186/s10020-021-00273-8

**Published:** 2021-01-28

**Authors:** Xunhu Gu, Hanjun Wu, Yuqin Xie, Lijun Xu, Xu Liu, Wei Wang

**Affiliations:** 1 Department of Neurology, The Second Affiliated Hospital of Nanchang University, No. 1 Minde Road, Nanchang, 330006 Jiangxi China; 2 Department of Laboratory Medicine, Jiangxi Health Vocational College, Nanchang, 330006 Jiangxi China

**Keywords:** Caspase-1, IL-1β, GluA1, Stargazin, AC-YVAD-CMK, Membrane transport, Alzheimer’s disease

## Abstract

**Background:**

Alzheimer's disease is a neurodegenerative disease. Previous study has reported that caspase-1/IL-1β is closely associated with Alzheimer's disease. However, the biological role of caspase-1/IL-1β in Alzheimer's disease has not been fully elucidated. This study aimed to explore the mechanism of action of caspase-1/IL-1β in Alzheimer's disease.

**Methods:**

Mouse hippocampal neurones were treated with Aβ_1-42_ to induce Alzheimer's disease cell model. APP/PS1 mice and Aβ_1-42_-induced hippocampal neurones were treated with AC-YVAD-CMK (caspase-1 inhibitor). Spatial learning and memory ability of mice were detected by morris water maze. Flow cytometry, TUNEL staining, Thioflavin S staining and immunohistochemistry were performed to examine apoptosis and senile plaque deposition. Enzyme linked immunosorbent assay and western blot were performed to assess the levels of protein or cytokines. Co-Immunoprecipitation was performed to verify the interaction between Stargazin and GluA1.

**Results:**

AC-YVAD-CMK treatment improved spatial learning and memory ability and reduced senile plaque deposition of APP/PS1 mice. Moreover, AC-YVAD-CMK promoted membrane transport of GluA1 in APP/PS1 mice. In vitro, Aβ_1-42_-induced hippocampal neurones exhibited an increase in apoptosis and a decrease in the membrane transport of GluA1, which was abolished by AC-YVAD-CMK treatment. In addition, Stargazin interacted with GluA1, which was repressed by caspase-1. Caspase-1/IL-1β inhibited membrane transport of GluA1 by inhibiting the interaction between Stargazin and GluA1.

**Conclusions:**

Our data demonstrate that caspase-1/IL-1β represses membrane transport of GluA1 by inhibiting the interaction between Stargazin in Alzheimer's disease. Thus, caspase-1/IL-1β may be a target for Alzheimer's disease treatment.

## Background

Alzheimer’s disease is a degenerative disease of the nervous system (Chen 2018). The main pathological features of Alzheimer's disease are the deposition of amyloid β-protein (Aβ) in the brain, neurofibrillary tangles, synaptic dysfunction and neuron loss (Kenney 2018). Alzheimer's disease is the most common type of senile dementia that occurs in old age. So far, the pathogenesis of Alzheimer's disease has not been fully elucidated, and there is no effective prevention and treatment method for Alzheimer's disease.

Synaptic plasticity is the neurophysiological basis of learning and memory. Under physiological or pathological conditions, synaptic plasticity is an important indicator of the changes of synaptic function and structure. Abnormal hippocampal synaptic plasticity has been considered as a key biological basis for cognitive dysfunction in Alzheimer's disease (Peineau et al. 2018). Previous study has confirmed that α-amino-3-hydroxy-5-methyl-4-isoxazolepropionic acid (AMPA) receptor is one of the three major receptors on the postsynaptic membrane that consists of GluA1-4. AMPA receptors are the main ionotropic glutamate receptors that mediate most of the rapid excitatory synaptic transmission in the vertebrate central nervous system. Accumulation studies have shown that abnormal AMPA receptor trafficking is an important feature of synaptic damage, and is closely related to synaptic plasticity and cognitive function in Alzheimer's disease (Guntupalli et al. 2016; Jurado 2017). For example, AMPA receptor subunits were down-regulated in hippocampus and entorhinal cortex of Alzheimer's disease patients, including GluA1, GluA2, and GluA2/3 (Armstrong and Ikonomovic 1996; Carter 2004). Almeida et al. have found that the total protein of GluA1 remains unchanged, while the surface expression of GluR1 is significantly reduced in APP mutant neurons (Almeida 2005). The study of Chang et al. has shown that the surface expression of GluA1 is down-regulated, and the AMPA receptor current is significantly reduced in the hippocampus of Alzheimer's disease mice (Chang 2006).

Previous study has reported that caspase-1 and its downstream inflammatory factor IL-1β are closely related to Alzheimer's disease (Heneka 2013). Caspase-1 mutation accelerates the progression of mild cognitive impairment to Alzheimer's disease (Pozueta 2011). Inhibition of caspase-1 improves hippocampal-dependent learning and memory in Alzheimer's disease mice (Flores 2018). Our previous study has also found that *Ginkgo biloba* extract EGb 761 improves the cognitive function of Alzheimer's disease mice by inhibiting the activity of caspase-1 (Liu 2015). Thus, caspase-1 may be an important molecular target for neuroprotection and intervention therapy for Alzheimer's disease. However, whether caspase-1 can regulate membrane transport disorder of AMPA receptors in Alzheimer's disease has not been reported yet. Our preliminary experiments have found that inhibition of caspase-1 enhances the surface expression of GluA1 in the hippocampus of APP/PS1 mice, suggesting that up-regulation of caspase-1 may participate in regulating membrane transport disorder of AMPA receptor.

Stargazin is a prosthetic protein of AMPA receptor, and Stargazin play a caucial role in regulating the membrane transport of GluA1 subunit of AMPA receptor in hippocampal neurons. Stargazin interacts with GluA1 subunit through the C-terminal PDZ domain, and then binds to PSD-95 to form a complex. After that, GluA1-containing AMPA receptors complex are quickly transported to the surface of synapsis (Shaikh 2016). The mutation or knockout at Starchazin PDZ domain functional sites affects membrane transport of AMPA receptor (Stein and Chetkovich 2010). Thus, this article aimed to explore whether caspase-1/IL-1β can affect GluA1 membrane transport by regulating the interaction between Stargazin and GluA1 in Alzheimer's disease.

## Materials and methods

### Experimental animals

Male APP/PS1 double transgenic Alzheimer's disease mic e (8-month-old) with C57BL/6J background and C57/BL6 mice (8-month-old, wild type, WT) were purchased from Beijing zhishan Co., Ltd (Beijing, China). All mice were housed under standardized conditions and received normal diet. APP/PS1 mice were randomly divided into 2 groups (n = 6): (1) APP/PS1-AC-YVAD-CMK group: APP/PS1 mice were intraperitoneally injected with AC-YVAD-CMK (caspase-1 inhibitor, 10 μg/kg, dissolved in 0.1% DMSO, Sigma-Aldrich, St. Louis, MO, USA) daily for 10 days; (2) APP/PS1 mice were intraperitoneally injected with equal 0.1% DMSO (Sigma-Aldrich) daily for 10 days. C57/BL6 mice (n = 6) were used as control. All protocols were authorized by the Ethics Committee of The Second Affiliated Hospital of Nanchang University ([2018] No. 085).

On the 11th day after modeling, the spatial learning and memory ability of mice were detected by morris water maze. Then, the mice were euthanized by cervical dislocation. The hippocampal tissues of mice were rapidly excised and snap-frozen in liquid nitrogen. The hippocampal tissues were stored at − 80 °C for further research.

### *Preparation of Aβ*_*1–42*_* oligomer*

Aβ_1–42_ oligomer was prepared as the previous reported (Yu 2018). In brief, Aβ_1–42_ (Sigma, St. Louis, MO, USA) was diluted with hexafluoroisopropanol (HFIP) to a concentration of 1 μg/μL, and then freeze-dried in the form of monomer. The Aβ_1–42_ peptide was subjected to vacuum condition to evaporate HFIP. For preparing Aβ_1–42_ oligomer, the peptide was resuspended in DMSO at a final concentration of 5 mmol/L. After that, the solution was incubated with phenol red-free F-12 media at 4 °C for 24 h. The supernatant was the Aβ_1–42_ oligomer, and stored at − 80 °C for further analysis.

### Cell culture

Mouse hippocampal neurones were purchased from public cell banks (ATCC, Manassas, VA, USA). The cells were cultured in Dulbecco's modified eagle medium: nutrient mixture F-12 (DMEM/F12) (Sangon Biotech, Shanghai, China) supplemented with 10% fetal bovine serum (FBS) and 1% penicillin/streptomycin. The cells were incubated in a humidified atmosphere at 37 °C and 5% CO_2_. The cells were cultured to the third generation for further analysis. Hippocampal neurones were treated with Aβ_1-42_ oligomer (10 μmol/L) for 24 h to induce Alzheimer's disease cell model. Then, the hippocampal neurones were treated with IL-1β (10 μg/L) or AC-YVAD-CMK (10 μmol/L) for 24 h.

### Cell transfection

Stargazin PDZ domain T321 mutation was subcloned into the vector pcDNA3.1 (Invitrogen, Carlsbad, CA, USA), generating the vector pcDNA3.1-Stargazin mutant. The negative control (NC) vector pcDNA3.1-NC was served as control (Ctrl). Hippocampal neurones were seeded into 96-well plate and cultured at 37 °C and 5% CO_2_ for 24 h. Then, hippocampal neurons (100 μL) were transfected with 0.2 μg pcDNA3.1-Stargazin mutant or pcDNA3.1-NC using 0.5 μL Lipofectamine 2000 (Invitrogen) following the manufacturer’s protocol. After 48 h of transfection, the transfected cells were collected and stored at − 20 °C for further analysis.

### Morris water maze

Spatial learning and memory ability of mice were detected by morris water maze as previous reported (Gu 2016). The circular water maze (122 cm diameter) was filled with water (21–23 °C) with a visible platform for 2 days. In the acquisition phase, mice were given a maximum of 60 s each trial to find the hidden platform and were required to remain seated on the platform for 10 s. After that, the mice were returned to their home cage. Subsequently, the platform was submerged 0.5 cm below the water surface, and mile was added into the water to make the platform invisible. The mice were allowed to swim freely in the maze until they found the hidden platform. The trail was carried out 4 times per day for 3 days with an interval of 1 h. On the sixth day, the mice were given up to 60 s to find the hidden platform. The latency period to find the hidden platform (escape latency) and the number of each mouse crossed the platform location in each trail were recorded using Videomex tracking system (Columbus Instruments, Columbus, OH, USA).

### Immunohistochemistry

Hippocampal tissues (subregions between DG and CA1) were fixed in 10% buffered formalin solution and then embedded in paraffin. Four-micron sections were obtained after deparaffin and rehydration. After that, the sections were treated with H_2_O_2_ for 5 min to eliminate the activity of endogenous peroxidases. The sections were incubated with primary antibody Aβ_1–42_ (Abcam, Cambridge, MA, USA) at 4 °C overnight. After washed with PBS for several times, the sections were incubated with horseradish peroxidase-secondary antibody at room temperature for 30 min. Subsequently, the sections were stained with diaminobenzidine and counterstained with hematoxylin. The senile plaque deposition in the hippocampal tissues was observed under a Nikon LV150N optical microscope (Nikon, Tokyo, Japan).

### Thioflavin S staining

Thioflavin S staining was performed to examine the senile plaque deposition in the hippocampal tissues (subregions between DG and CA1) of mice. Hippocampal tissues were fixed in 4% paraformaldehyde and embedded in paraffin. The sections with 30 μm were obtained after deparaffin and rehydration. The sections were stained with 1% thioflavin-S for 5 min. After that, the sections were washed with 70% ethanol and distilled water. Images of sections were captured using BX43 fluorescence microscope (Olympus, Tokyo, Japan). Three same sections were analyzed per mouse in the hippocampal tissues. The images were analyzed using Image Pro Plus 6 Media Cybernetics (Silver Spring, Maryland, USA).

### TdT-mediated dUTP nick-end labeling (TUNEL) assay

Hippocampal tissues (subregions CA1) were fixed in 4% paraformaldehyde and embedded in paraffin. Five-micron sections were obtained after deparaffin and rehydration. To assess the levels of apoptosis of hippocampal neuron, paraffin sections were stained using TUNEL apoptosis assay kit (Solarbio, Beijing, China) according to the manufacturer’s protocol. The stained sections were then observed under the Nikon LV150N optical microscope (Nikon, Tokyo, Japan).

### Western blot (WB)

Total protein was extracted from hippocampal neurones or hippocampal tissues using Tissue or Cell Total Protein Extraction Kit (Sangon Biotech). Cell-surface and cytosol protein were extracted from hippocampal neurones using Membrane and Cytosol Protein Extraction Kit (Beyotime Biotechnology, Shanghai, China). The concentration of proteins was assessed using BCA Protein Assay Kit (Sangon Biotech) as the protocol described. Equivalent protein (25 μg) from different samples was separated by 10% SDS-PAGE protein electrophoresis, following by transformation onto PVDF membranes (Merck Millipore, Billerica, MA, USA). The membranes were blocked with 5% skim milk at room temperature for 2 h, and then incubated with the primary antibodies, caspase-1, GluA1 (surface, S), GluA1 (intracellular, I) and Stargazin (1:1000, Abcam) at 4 °C overnight. After the membranes were washed with TBST for several times, horseradish peroxidase-conjugate second antibody (1:1000, Abcam) was incubated with the membranes. β-actin antibody (1:1000, Abcam) was used as a reference protein for normalization. The gray levels of the protein bands were examined by Image J software.

### Enzyme linked immunosorbent assay (ELISA)

The levels of IL-1β in the hippocampal tissues or culture supernatant of hippocampal neurones were assessed using IL-1β ELISA Kit (MultiSciences, Hangzhou, China) according to the manufacturers' instruction. The optical density values of samples were detected using enzyme-labeled instrument (Thermo Fisher Scientific, Waltham, MA, USA).

### Flow cytometry

Flow cytometry was performed to detect the apoptosis of hippocampal neurones. Hippocampal neurones were seeded into 96-well plate and treated with Aβ_1-42_ oligomer (10 μmol/L), IL-1β (10 μg/L) or AC-YVAD-CMK (10 μmol/L) for 24 h. After that, the cells were collected and washed with pre-cooled PBS for 2 times. Cells were then resuspended in the Annexin V Binding buffer. The cell suspension was dyed with Annexin V-FITC and PI and plunged into darkness at room temperature for 15 min. Then, the cell suspension was mixed with Annexin V Binding buffer and put on ice. The apoptosis rate of cells was determined by flow cytometry in an hour. The assay was performed according to the instruction of Annexin V-FITC/PI Cell Apoptosis Detection Kit (TransGen Biotech, Beijing, China).

### Co-Immunoprecipitation (Co-IP)

Co-IP assay was performed to verify the interaction between Stargazin and GluA1 in hippocampal neurones. Hippocampal neurones were co-transfected with pTT5-Stargazin-His and pTT5-GluA1-Flag. After 48 h of transfection, the cell lysates were harvested by centrifugation at 5000 rpm for 10 min, and then incubated with Flag-tagged antibody (Proteintech, Wuhan, China) at 4 °C overnight. Furthermore, Protein A/G Plus-Agarose (Santa Cruz Biotechnology, Santa Cruz, CA, USA) was incubated with the mixture at 4 °C for 6 h. Immunoprecipitates were collected by centrifugation at 3000 rpm for 5 min, and washed with pre-cooled PBS for several times. After that, the immunoprecipitates were identified by WB using the primary antibodies, GluA1 and Stargazin.

### Statistical analysis

All experiments were independently repeated at least 3 times. All values were exhibited as mean ± standard deviation and analyzed by SPSS 22.0 statistical software (IBM, Armonk, NY, USA). For comparison of two groups, a two-tailed Student’s t test was used. Comparison of multiple groups was made using one-way ANOVA. Difference was considered statistically significant at *P* < 0.05.

## Results

### AC-YVAD-CMK effectively improved spatial learning and memory ability and reduced senile plaque deposition of APP/PS1 mice

**Fig. 1:**
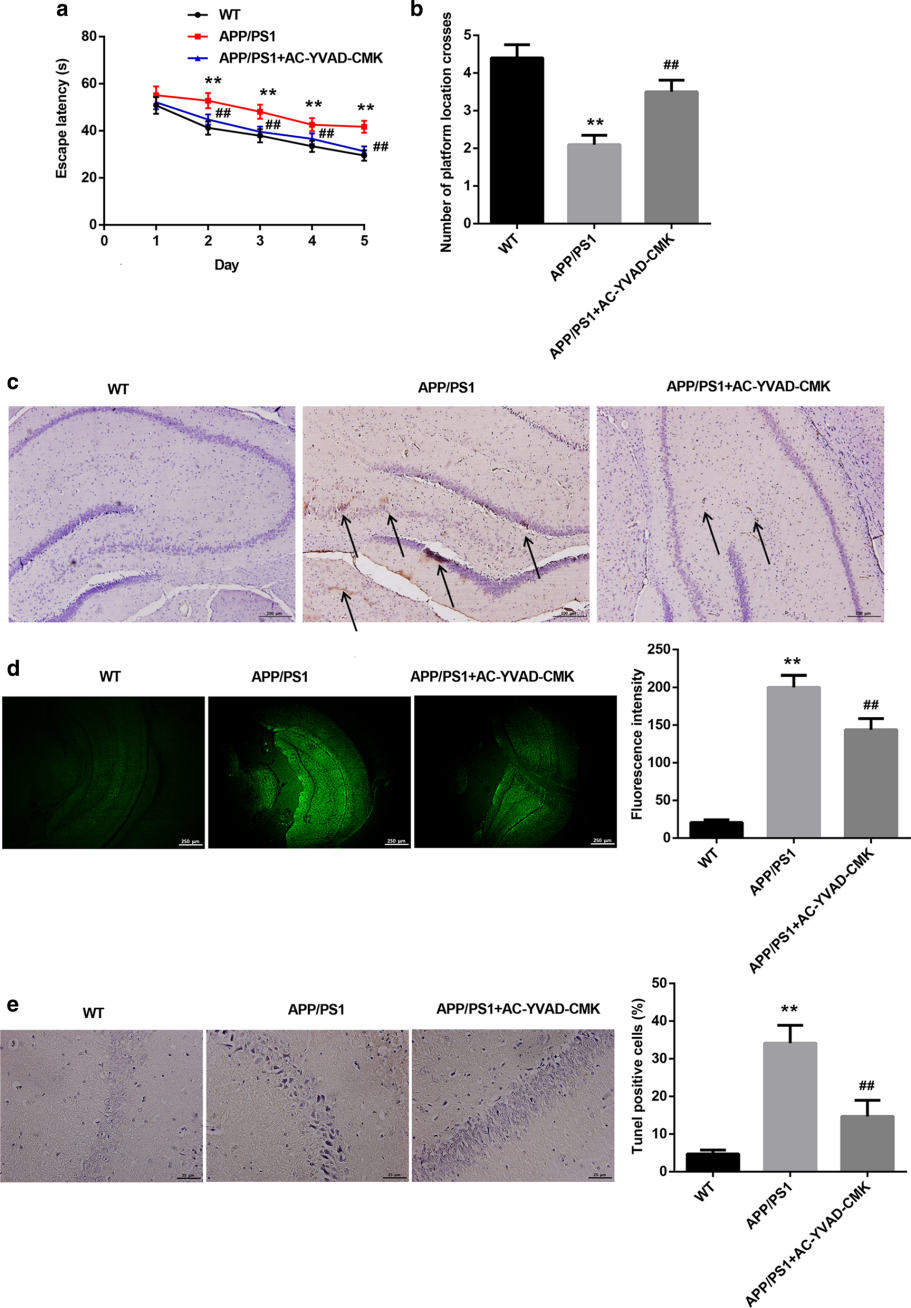
AC-YVAD-CMK improved spatial learning and memory ability and reduced senile plaque deposition of APP/PS1 mice. APP/PS1 mice were intraperitoneally injected with AC-YVAD-CMK or DMSO. WT C57/BL6 mice were served as control. **a**, **b** Spatial learning and memory ability of mice were detected by morris water maze. **c**, **d** Immunohistochemistry and Thioflavin S staining were performed to examine the senile plaque deposition in the hippocampal tissues (subregions between DG and CA1) of mice. **e** TUNEL staining was performed to assess apoptosis in hippocampal neurones of mice. The arrows indicated the Aβ_1-42_. (One-way ANOVA; **P < 0.01 vs. WT group; ^##^*P* < 0.01 vs. APP/PS1 group.)

We initially inhibited the activity of caspase-1 by AC-YVAD-CMK, and determined the influence of caspase-1 inhibition on the spatial learning and memory ability of APP/PS1 mice. The results of morris water maze showed that APP/PS1 mice exhibited longer escape latency as compared with WT mice. AC-YVAD-CMK treatment reduced escape latency of APP/PS1 mice (Fig. 1a). The number of platform location crosses of APP/PS1 mice was less than WT mice, while AC-YVAD-CMK treatment significantly enhanced the number of platform location crosses of APP/PS1 mice (Fig. 1b). Subsequently, we performed immunohistochemistry and Thioflavin S staining to examine the senile plaque deposition in the hippocampal tissues of mice. APP/PS1 mice displayed an increase of senile plaque deposition in the hippocampal tissues (subregions between DG and CA1), which was effectively reduced by AC-YVAD-CMK treatment (Fig. 1c, d). Moreover, TUNEL staining data showed that the apoptosis of hippocampal neurones (subregions CA1) was obviously increased in APP/PS1 mice with respect to WT mice. AC-YVAD-CMK treatment led to a decrease in apoptosis of hippocampal neurones (subregions CA1) in APP/PS1 mice (Fig. 1e). Taken together, these data suggested that the caspase-1 inhibitor, AC-YVAD-CMK, effectively improved spatial learning and memory ability and reduced senile plaque deposition of APP/PS1 mice.

### AC-YVAD-CMK promoted membrane transport of GluA1 in APP/PS1 mice

**Fig. 2:**
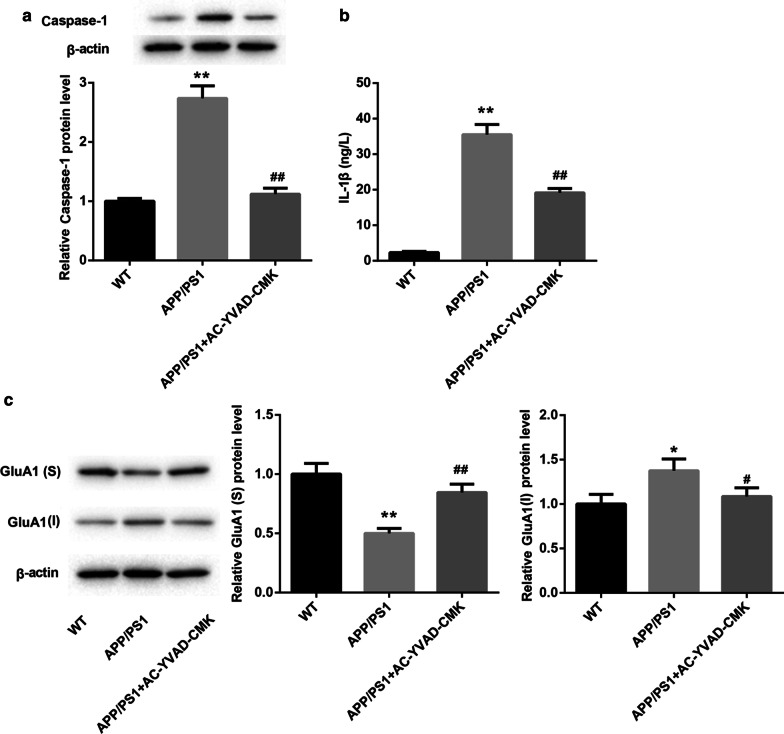
AC-YVAD-CMK promoted membrane transport of GluA1 in APP/PS1 mice. APP/PS1 mice were intraperitoneally injected with AC-YVAD-CMK or DMSO. WT C57/BL6 mice were served as control. **a** WB was performed to detect the expression of caspase-1 in the hippocampal tissues of mice. **b** ELISA was performed to assess the levels of IL-1β in the hippocampal tissues of mice. **c** WB was performed to estimate the expression of GluA1 in the hippocampal tissues of mice. S: surface; I: intracellular. (One-way ANOVA; *P < 0.05, **P < 0.01 vs. WT group; ^#^*P* < 0.05, ^##^*P* < 0.01 vs. APP/PS1 group.)

We further explored whether AC-YVAD-CMK can affect the membrane transport of GluA1 in APP/PS1 mice. WB data revealed that APP/PS1 mice displayed an increase of caspase-1 expression as compared with WT mice. AC-YVAD-CMK treatment caused a down-regulation of caspase-1 in the hippocampal tissues of APP/PS1 mice (Fig. 2a). Furthermore, we assessed the levels of IL-1β in the hippocampal tissues of mice by ELISA, showing that the levels of IL-1β in APP/PS1 mice were higher than that in WT mice. The levels of IL-1β were severely decreased in the hippocampal tissues of APP/PS1 mice in the presence of AC-YVAD-CMK treatment (Fig. 2b). We also found that compared with WT mice, APP/PS1 mice exhibited a decrease of GluA1 surface expression and an increase of GluA1 intracellular expression. However, AC-YVAD-CMK treatment promoted GluA1 surface expression and inhibited GluA1 intracellular expression in APP/PS1 mice (Fig. 2c). Therefore, these findings confirmed that AC-YVAD-CMK promoted membrane transport of GluA1 in APP/PS1 mice.

### *Caspase-1/IL-1β promoted apoptosis and inhibited membrane transport of GluA1 in Aβ*_*1-42*_*-induced hippocampal neurones*

**Fig. 3:**
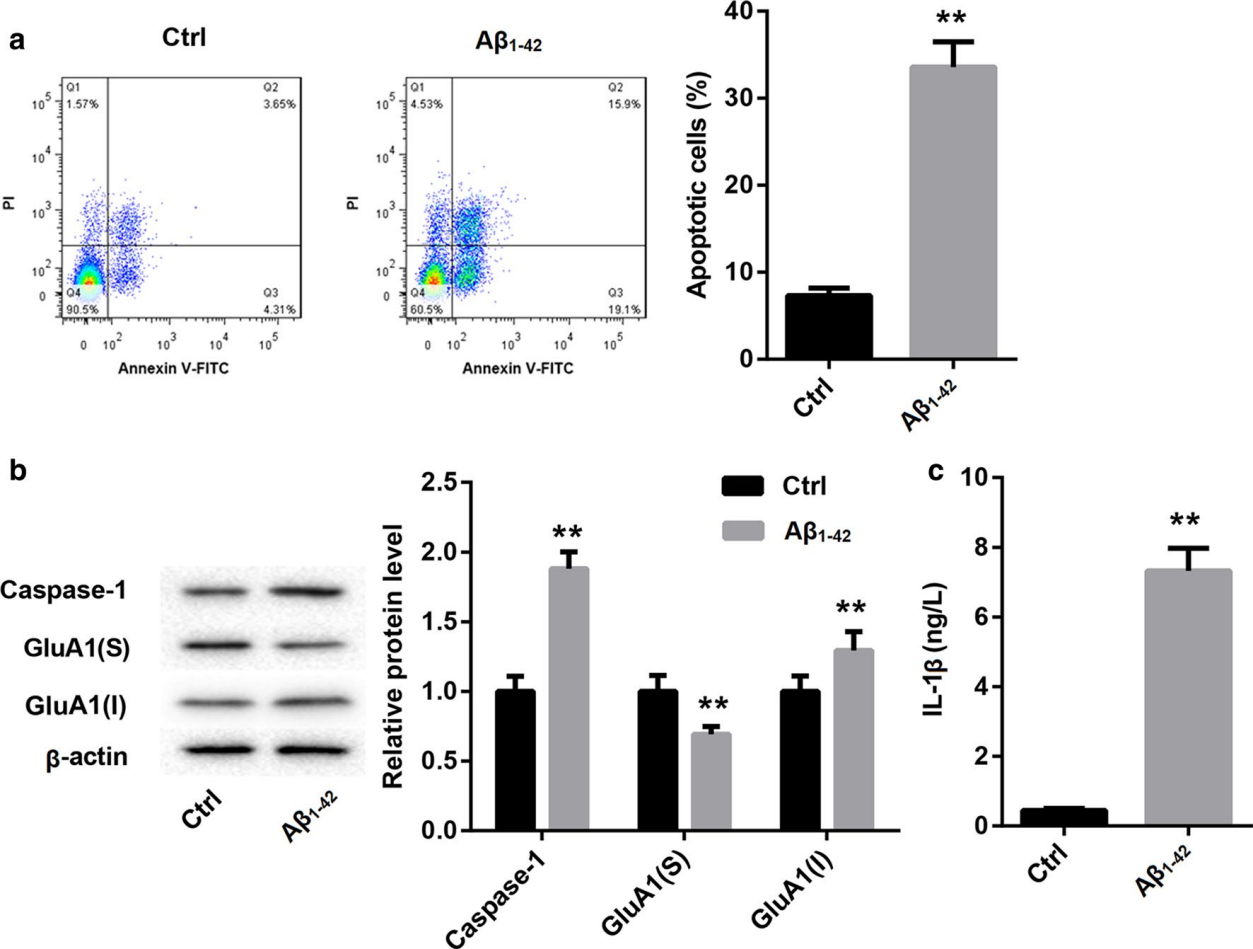
Aβ_1-42_-induced hippocampal neurones exhibited an increase in apoptosis and a decrease in the membrane transport of GluA1. Hippocampal neurones were treated with Aβ_1-42_ oligomer (10 μmol/L) for 24 h. Normal hippocampal neurones were served as control. **a** The apoptosis of hippocampal neurones was examined by flow cytometry. **b** WB was performed to detect the expression of caspase-1 and GluA1 in the hippocampal neurones. **c** ELISA was performed to assess the levels of IL-1β in the hippocampal neurones. S: surface; I: intracellular. (Student’s t test; *P < 0.05, **P < 0.01 vs. Ctrl group.)

**Fig. 4:**
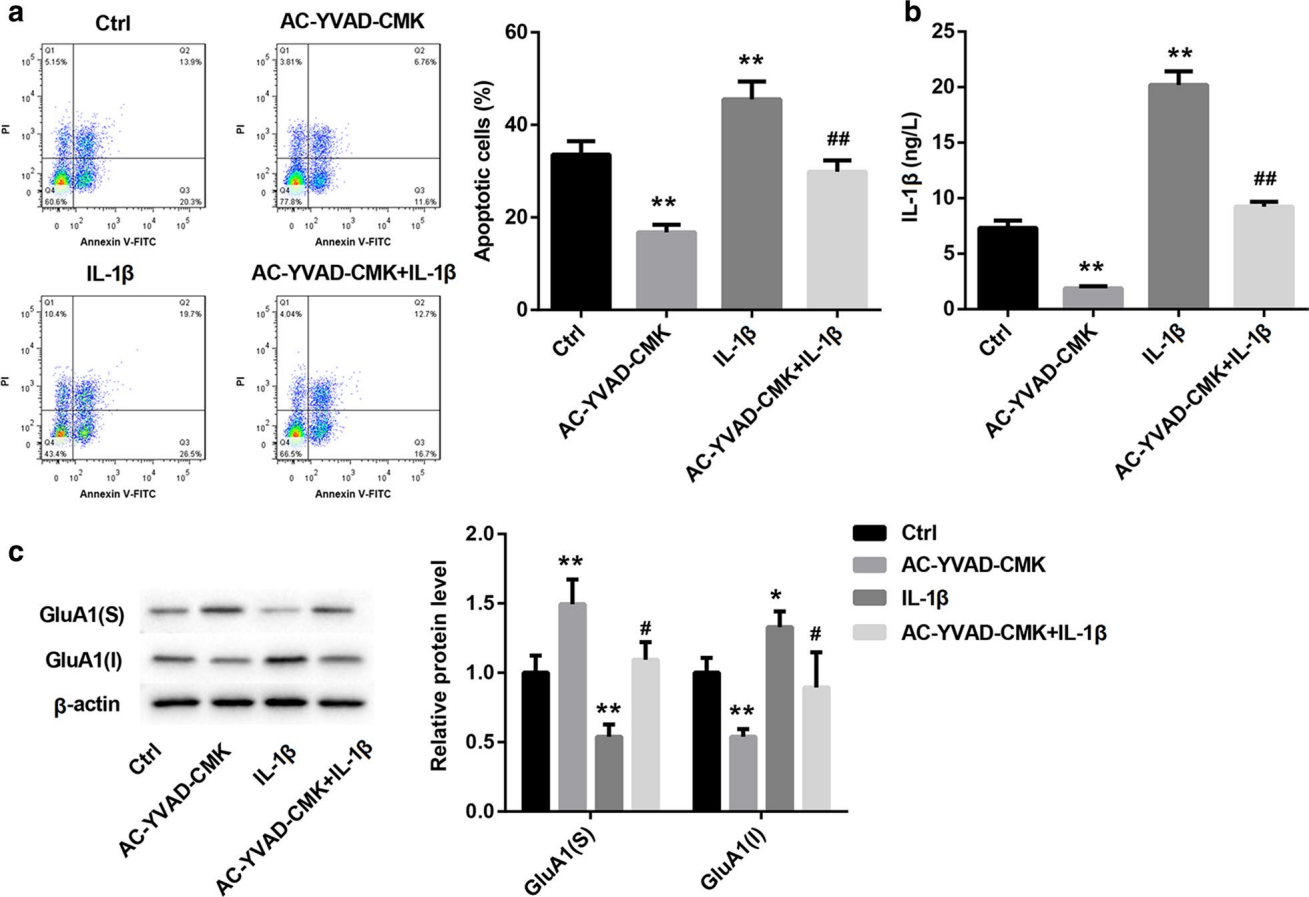
Caspase-1/IL-1β promoted apoptosis and inhibited membrane transport of GluA1 in Aβ_1-42_-induced hippocampal neurones. Hippocampal neurones were treated with Aβ_1-42_ oligomer (10 μmol/L) for 24 h, and then treated with IL-1β (10 μg/L) or AC-YVAD-CMK (10 μmol/L) for 24 h. Aβ_1-42_-treated hippocampal neurones served as control. **a** The apoptosis of hippocampal neurones was examined by flow cytometry. **b** ELISA was performed to assess the levels of IL-1β in the hippocampal neurones. **c** WB was performed to detect the expression of GluA1 in the hippocampal neurones. S: surface; I: intracellular. (One-way ANOVA; *P < 0.05, **P < 0.01 vs. Ctrl group; ^#^*P* < 0.05, ^##^*P* < 0.01 vs. AC-YVAD-CMK group.)

Next, we determined whether caspase-1/IL-1β can regulate apoptosis and membrane transport of GluA1 in Aβ_1-42_-induced hippocampal neurones. The data of flow cytometry revealed that Aβ_1-42_-induced hippocampal neurones displayed a boost in apoptosis as compared with Ctrl group (Fig. 3a). Subsequently, WB was performed to estimate the expression of caspase-1 and the surface and intracellular expression of GluA1 in the hippocampal neurones. Aβ_1-42_ treatment enhanced the expression of caspase-1 and the intracellular expression of GluA1, whereas Aβ_1-42_ treatment led to a down-regulation of surface GluA1 in the hippocampal neurons (Fig. 3b). Moreover, Aβ_1-42_ treatment significantly enhanced the levels of IL-1β in the hippocampal neurons (Fig. 3c). In addition, Aβ_1-42_-induced hippocampal neurones were treated with AC-YVAD-CMK or combined with IL-1β. Flow cytometry results were showed that AC-YVAD-CMK treatment repressed apoptosis of Aβ_1-42_-induced hippocampal neurones, whereas IL-1β treatment promoted apoptosis of Aβ_1-42_-induced hippocampal neurones. However, the apoptosis of Aβ_1-42_-induced hippocampal neurones was enhanced in the presence of AC-YVAD-CMK combined with IL-1β (Fig. 4a). Then, ELISA was performed to assess the levels of IL-1β in the hippocampal neurones. The levels of IL-1β in Aβ_1-42_-induced hippocampal neurones were suppressed by AC-YVAD-CMK treatment, and enhanced by IL-1β treatment. The influence conferred by AC-YVAD-CMK treatment was abolished by IL-1β treatment (Fig. 4b). Furthermore, AC-YVAD-CMK treatment caused an increase of GluA1 surface expression and a decrease of GluA1 intracellular expression in Aβ_1-42_-induced hippocampal neurones. However, IL-1β treatment inhibited GluA1 surface expression and enhanced GluA1 intracellular expression in the Aβ_1-42_-induced hippocampal neurons. IL-1β treatment impaired the influence of AC-YVAD-CMK treatment on GluA1 surface and intracellular expression in the Aβ_1-42_-induced hippocampal neurones (Fig. 4c). Thus, these results demonstrated that caspase-1/IL-1β promoted apoptosis and inhibited membrane transport of GluA1 in Aβ_1-42_-induced hippocampal neurones.

### Caspase-1/IL-1β inhibited membrane transport of GluA1 by inhibiting the interaction between Stargazin and GluA1

**Fig. 5:**
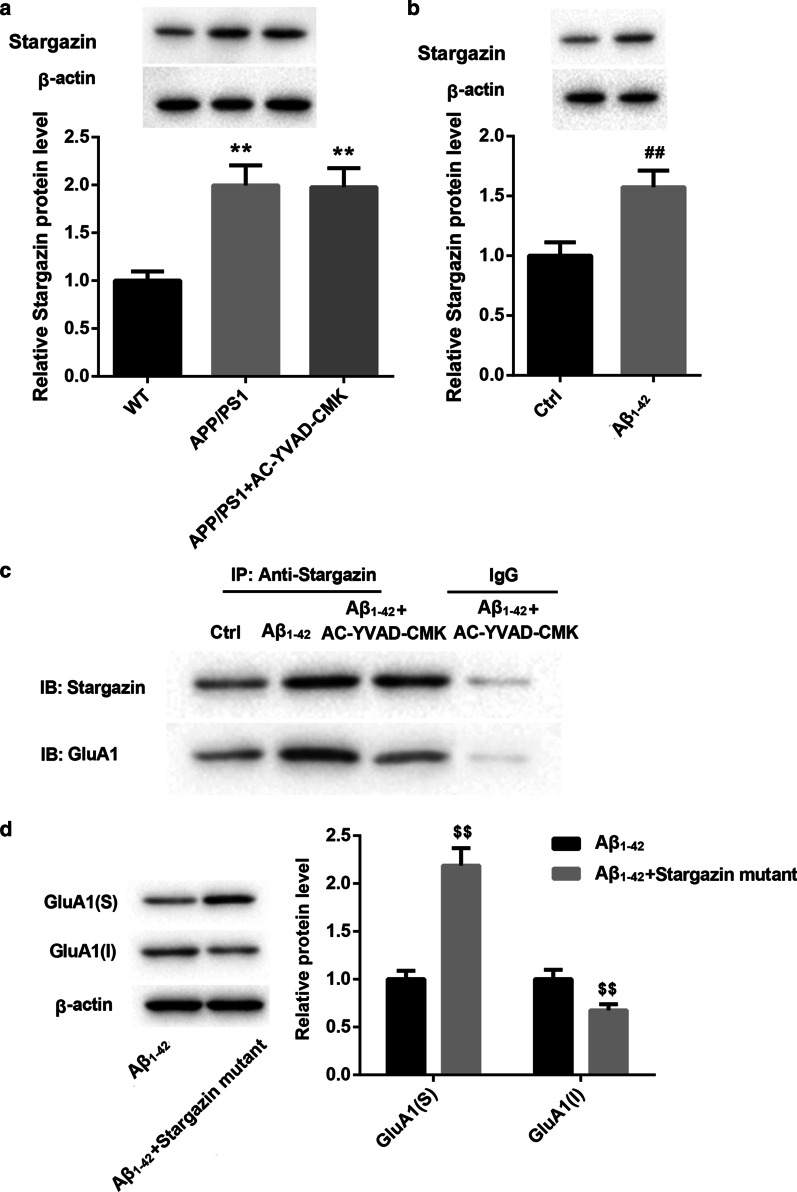
Caspase-1/IL-1β inhibited membrane transport of GluA1 by inhibiting the interaction between Stargazin and GluA1. APP/PS1 mice were intraperitoneally injected with AC-YVAD-CMK or DMSO. WT C57/BL6 mice were served as control. **a** WB was performed to detect the expression of Stargazin in the hippocampal tissues of mice (One-way ANOVA). Hippocampal neurones were treated with Aβ_1-42_ oligomer (10 μmol/L) for 24 h. Normal hippocampal neurones were served as control. **b** WB was performed to assess the expression of Stargazin in the hippocampal neuron cells (Student’s t test). **c** Co-IP assay was performed to verify the interaction between Stargazin and GluA1 in hippocampal neurones. Hippocampal neurones were transfected with pcDNA3.1-Stargazin mutant or pcDNA3.1-NC. Then, the modified hippocampal neurones were treated with Aβ_1-42_ oligomer (10 μmol/L) combined with AC-YVAD-CMK (10 μmol/L) for 24 h. **d** WB was performed to detect the expression of GluA1 in the modified hippocampal neurones (Student’s t test). S: surface; I: intracellular. **P < 0.01 vs. WT group; ^##^*P* < 0.01 vs. Ctrl group; ^$$^*P* < 0.01 vs. Aβ_1-42_ group

Finally, we explored the mechanism of action of caspase-1/IL-1β in inhibiting the membrane transport of GluA1. WB data showed that APP/PS1 mice and AC-YVAD-CMK-treated APP/PS1 mice all exhibited an up-regulation of Stargazin (Fig. 5a). Stargazin was also highly expressed in the Aβ_1-42_-induced hippocampal neurones (Fig. 5b). Subsequently, we performed Co-IP to verify the relationship between Stargazin and GluA1, showing that Stargazin interacted with GluA1 in the Aβ_1-42_-induced hippocampal neurons. However, caspase-1 inhibited the interaction between Stargazin and GluA1 in the Aβ_1-42_-induced hippocampal neurones (Fig. 5c). Furthermore, hippocampal neurones were transfected with pcDNA3.1-Stargazin mutant to induce Stargazin mutant overexpression in hippocampal neurons. We found that Stargazin mutant overexpression led to an increase of GluA1 surface expression, whereas Stargazin mutant up-regulation inhibited GluA1 intracellular expression in the Aβ_1-42_-induced hippocampal neurones (Fig. 5d). Thus, these data demonstrated that caspase-1/IL-1β inhibited membrane transport of GluA1 by inhibiting the interaction between Stargazin and GluA1.

## Discussion

Caspase-1/IL-1β participates in the development of Alzheimer's disease. Chronic cerebral hypoperfusion accelerates Alzheimer's disease by enhancing NLRP3 inflammasome, and activating caspase-1 and IL-1β in hippocampus and thalamus of mice (Shang 2019). TXNIP up-regulation sustains neurodegeneration by activating NLRP3 inflammasome, and enhancing the expression of caspase-1 and IL-1β in the brains of Alzheimer's disease patients (Li 2019). NLRC4 inflammasome induces neuroinflammation and contributes to memory impairment in Alzheimer's disease rats through the activation of caspase-1 and IL-1β (Saadi 2020). Consistently, we also found that the levels of caspase-1 and IL-1β were significantly enhanced in the APP/PS1 mice, which was effectively abolished by AC-YVAD-CMK treatment. Thus, we used AC-YVAD-CMK to inhibit activity of caspase-1/IL-1β to further explore the mechanism of action of caspase-1/IL-1β in the pathogenesis of Alzheimer's disease. We found that AC-YVAD-CMK significantly improved spatial learning and memory ability, and reduced senile plaque deposition and apoptosis of hippocampal neurones in APP/PS1 mice. Thus, these data suggested that AC-YVAD-CMK may improve Alzheimer's disease of APP/PS1 mice by repressing caspase-1 activity. In addition, Lin et al. have confirmed that AC-YVAD-CMK treatment improves neurological function and some degree of limb movement and reduces the levels of inflammatory factors in intracerebral hemorrhage mice (Lin 2018). Therefore, we speculated that AC-YVAD-CMK treatment may improve some degree of limb movement in APP/PS1 mice. This speculation still needs further researched to verify.

GluA1 is an important subtype of AMPA receptor, and is a key component in neurocognitive networks (Zhao 2018). GluA1 participates in the transport and integration of AMPA receptors in the synaptic membranes. GluA1 up-regulation and phosphorylation are closely related to the treatment of Alzheimer's disease, schizophrenia, depression, and chronic drug addiction (Zhang and Abdullah 2013). In the development of Alzheimer's disease, GluA1 ubiquitination mediates the loss of surface AMPA receptors induced by Aβ (Guntupalli 2017). Perfluorooctane sulfonate exposure induces cognitive function disorder by inhibiting the expression of GluA1 and GluA2 in the membrane of primary hippocampal neurons (Zhang 2019). Consistently, our study also confirmed that APP/PS1 mice exhibited a down-regulation of surface GluA1 and an up-regulation of intracellular GluA1 in the hippocampal tissues, which was effectively rescued by AC-YVAD-CMK treatment. Thus, these data suggested that AC-YVAD-CMK treatment may improve cognitive function of APP/PS1 mice by promting membrane transport of GluA1. In addition, our in vitro experiments further demonstrated that AC-YVAD-CMK treatment inhibited apoptosis and promoted membrane transport of GluA1 in the Aβ_1-42_-treated hippocampal neurones by inhibiting caspase-1/IL-1β activity.

Stargazin plays a crucial role in GluA1-containing AMPA receptors trafficking (Shaikh 2016). The abnormal association between Stargazin and AMPA subunits affects the forward trafficking or synaptic stability of GluA1-containing AMPA reporters, which is closely associated with schizophrenia (Benesh et al. 2020). During synaptic downscaling, Stargazin dephosphorylation is related to the increased surface mobility of Stargazin and synaptic GluA1 (Louros et al. 2018). In the present research, we determined whether caspase-1/IL-1β can regulate membrane transport of GluA1 through Stargazin. We found that Stargazin interacted with GluA1 and promoted membrane transport of GluA1 in Aβ_1-42_-induced hippocampal neurones. Therefore, caspase-1/IL-1β inhibited membrane transport of GluA1 by inhibiting the interaction between Stargazin and GluA1.

## Conclusion

In conclusion, our data demonstrate that caspase-1/IL-1β participates in pathogenesis of Alzheimer's disease. Caspase-1/IL-1β represses membrane transport of GluA1 by inhibiting the interaction between Stargazin in Alzheimer's disease. Thus, our research provides a theoretical basis for the pathogenesis of Alzheimer's disease, and caspase-1/IL-1β may be a target for Alzheimer's disease treatment.

## Data Availability

The datasets used and/or analysed during the current study are available from the corresponding author on reasonable request.

## Abbreviations

AMPA: α-Amino-3-hydroxy-5-methyl-4-isoxazolepropionic acid
Aβ: Amyloid β-protein
Co-IP: Co-immunoprecipitation
Ctrl: Control
DMEM/F12: Dulbecco's modified eagle medium: nutrient mixture F-12
ELISA: Enzyme linked immunosorbent assay
FBS: Fetal bovine serum
NC: Negative control
TUNEL: TdT-mediated dUTP nick-end labeling
WB: Western blot
WT: Wild type

## References

[CR1] Almeida CG (2005). Beta-amyloid accumulation in APP mutant neurons reduces PSD-95 and GluR1 in synapses. Neurobiology of disease.

[CR2] Armstrong DM, Ikonomovic MD (1996). AMPA-selective glutamate receptor subtype immunoreactivity in the hippocampal dentate gyrus of patients with Alzheimer disease. Evidence for hippocampal plasticity. Mol Chem Neuropathol.

[CR3] Benesh JL, Mueller TM, Meador-Woodruff JH (2020). AMPA receptor subunit localization in schizophrenia anterior cingulate cortex. Schizophrenia Res.

[CR4] Carter TL (2004). Differential preservation of AMPA receptor subunits in the hippocampi of Alzheimer's disease patients according to Braak stage. Exp Neurol.

[CR5] Chang EH (2006). AMPA receptor downscaling at the onset of Alzheimer's disease pathology in double knockin mice. Proc Natl Acad Sci USA.

[CR6] Chen YG (2018). Research progress in the pathogenesis of Alzheimer's disease. Chin Med J.

[CR7] Flores J (2018). Caspase-1 inhibition alleviates cognitive impairment and neuropathology in an Alzheimer's disease mouse model. Nat Commun.

[CR8] Gu XH (2016). The flavonoid baicalein rescues synaptic plasticity and memory deficits in a mouse model of Alzheimer's disease. Behav Brain Res.

[CR9] Guntupalli S (2017). GluA1 subunit ubiquitination mediates amyloid-β-induced loss of surface α-amino-3-hydroxy-5-methyl-4-isoxazolepropionic acid (AMPA) receptors. J Biol Chem.

[CR10] Guntupalli S, Widagdo J, Anggono V (2016). Amyloid-β-induced dysregulation of AMPA receptor trafficking. Neural Plast.

[CR11] Heneka MT (2013). NLRP3 is activated in Alzheimer's disease and contributes to pathology in APP/PS1 mice. Nature.

[CR12] Jurado S (2017). AMPA receptor trafficking in natural and pathological aging. Front Mol Neurosci.

[CR13] Kenney K (2018). Dementia after moderate-severe traumatic brain injury: coexistence of multiple proteinopathies. J Neuropathol Exp Neurol.

[CR14] Li L (2019). Thioredoxin-Interacting Protein (TXNIP) Associated NLRP3 Inflammasome Activation in Human Alzheimer's Disease Brain. JAD.

[CR15] Lin X (2018). AC-YVAD-CMK inhibits pyroptosis and improves functional outcome after intracerebral hemorrhage. Biomed Res Int.

[CR16] Liu X (2015). Long-term treatment with Ginkgo biloba extract EGb 761 improves symptoms and pathology in a transgenic mouse model of Alzheimer's disease. Brain Behav Immun.

[CR17] Louros SR, Caldeira GL, Carvalho AL (2018). Stargazin dephosphorylation mediates homeostatic synaptic downscaling of excitatory synapses. Front Mol Neurosci.

[CR18] Peineau S, Rabiant K, Pierrefiche O, Potier B (2018). Synaptic plasticity modulation by circulating peptides and metaplasticity: Involvement in Alzheimer's disease. Pharmacol Res.

[CR19] Pozueta A (2011). Genetic variation in caspase-1 as predictor of accelerated progression from mild cognitive impairment to Alzheimer's disease. J Neurol.

[CR20] Saadi M (2020). Involvement of NLRC4 inflammasome through caspase-1 and IL-1β augments neuroinflammation and contributes to memory impairment in an experimental model of Alzheimer's like disease. Brain Res Bull.

[CR21] Shaikh SA (2016). Stargazin modulation of AMPA receptors. Cell Rep.

[CR22] Shang J (2019). Acceleration of NLRP3 inflammasome by chronic cerebral hypoperfusion in Alzheimer's disease model mouse. Neurosci Res.

[CR23] Stein EL, Chetkovich DM (2010). Regulation of stargazin synaptic trafficking by C-terminal PDZ ligand phosphorylation in bidirectional synaptic plasticity. J Neurochem.

[CR24] Yu X (2018). Magnesium ions inhibit the expression of tumor necrosis factor α and the activity of γ-secretase in a β-amyloid protein-dependent mechanism in APP/PS1 transgenic mice. Front Mol Neurosci.

[CR25] Zhang Q (2019). Developmental perfluorooctane sulfonate exposure inhibits long-term potentiation by affecting AMPA receptor trafficking. Toxicology.

[CR26] Zhang J, Abdullah JM (2013). The role of GluA1 in central nervous system disorders. Rev Neurosci.

[CR27] Zhao LX (2018). M1 muscarinic receptor facilitates cognitive function by interplay with AMPA receptor GluA1 subunit. FASEB journal.

